# P-737. Prescribing Patterns of Doxycycline for Post-Exposure Prophylaxis and Trends in Sexually Transmitted Infections at a Large Academic HIV Clinic

**DOI:** 10.1093/ofid/ofaf695.948

**Published:** 2026-01-11

**Authors:** Rachael Pellegrino, Matthew Lokant, Nishant Patel, Christina Vojtek, Priya Jagadeesan, Andrea Ito, Richard M Merkhofer, Kaitlyn Reasoner, Michael Zou, Austin Katona, Megan Turner, Jim Zhang, Chris Terndrup, Sean Kelly, Casey Smiley

**Affiliations:** Vanderbilt University Medical Center, Nashville, TN; VUMC, Nashville, Tennessee; University of Louisville School of Medicine; Vanderbilt University Medical Center, Nashville, TN; Vanderbilt University Medical Center, Nashville, TN; 4Vanderbilt University, Nashville, Tennessee; Vanderbilt University Medical Center, Nashville, TN; Vanderbilt University Medical Center, Nashville, TN; 4Vanderbilt University, Nashville, Tennessee; Vanderbilt University Medical Center, Nashville, TN; Vanderbilt University Medical Center, Nashville, TN; Vanderbilt University Medical Center, Nashville, TN; Vanderbilt University Medical Center, Nashville, TN; Vanderbilt University Medical Center, Nashville, TN; Vanderbilt University Medical Center, Nashville, TN

## Abstract

**Background:**

Evaluation of real-world uptake and effectiveness of doxycycline post-exposure prophylaxis (DoxyPEP) to prevent sexually transmitted infections (STI) in people with HIV (PWH) is limited, especially in the Southeastern US. The Vanderbilt Comprehensive Care Clinic (VCCC) implemented a DoxyPEP protocol in November 2023, and we aimed to assess prescribing patterns and changes in STIs.

**Figure:**
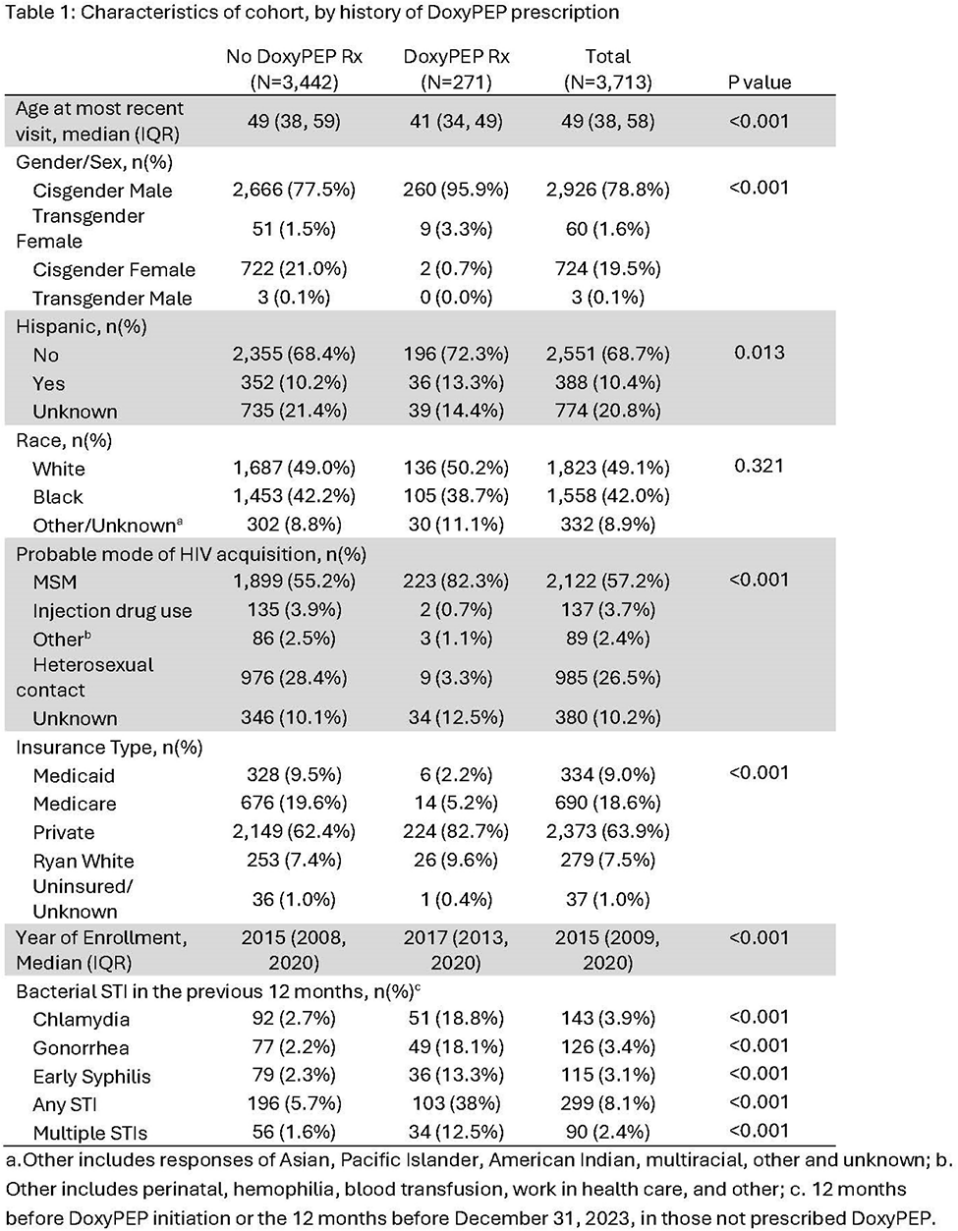

**Figure:**
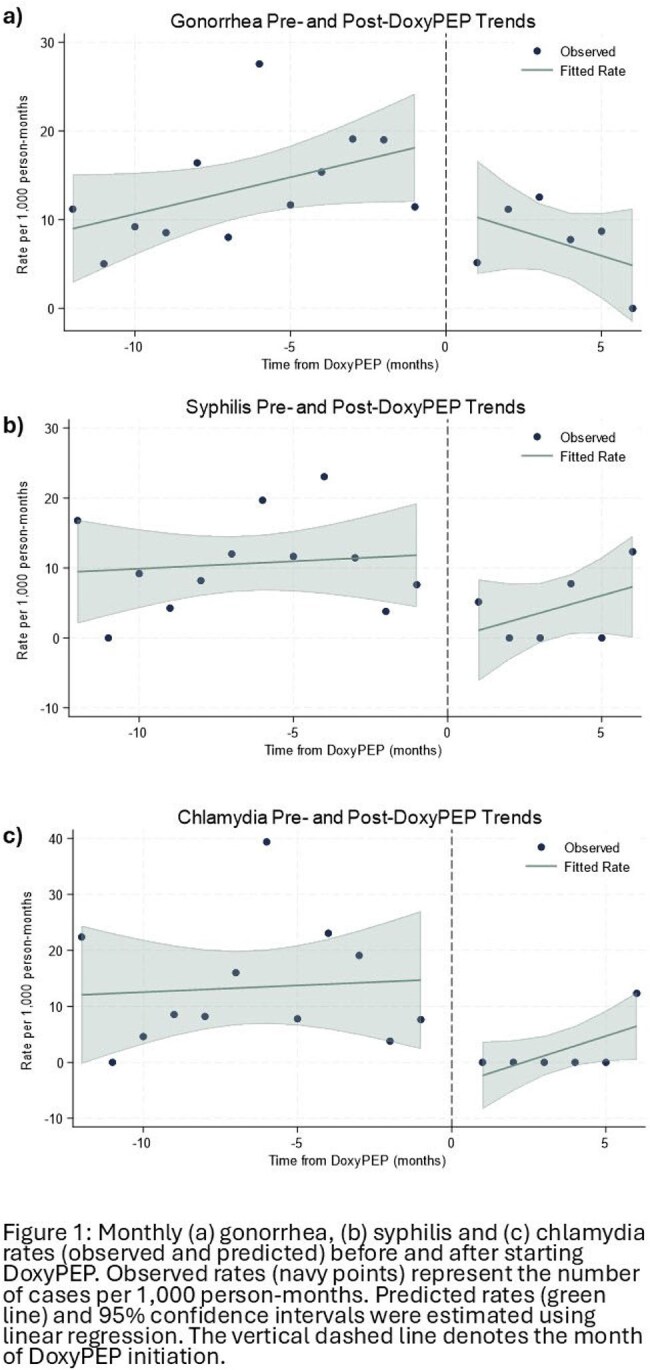

**Methods:**

This retrospective cohort study included adults who receive HIV care at the VCCC. New DoxyPEP prescriptions were assessed from 1/1/2023-7/31/2024. Baseline characteristics for those prescribed and not prescribed DoxyPEP were compared using descriptive statistics. We determined the monthly STIs (chlamydia, gonorrhea, and early syphilis) and person-time under observation for the 12 months before and 6 months after DoxyPEP initiation (excluding the time of initiation). To calculate incidence rate ratios (IRR) comparing STI rates before and after DoxyPEP, we used a generalized linear model with a Poisson distribution, log link, and the natural log of person-months included as an offset and a binary variable indicating DoxyPEP status. Predicted and observed STI rates were visualized over time with linear regression.

**Results:**

Overall, 3,713 PWH were included in the cohort and 271 (7%) individuals were prescribed DoxyPEP during the study period. PWH prescribed DoxyPEP were more likely to be younger, male sex, privately insured, and to have had an STI in the prior 12 months but there were no differences by race (Table 1). For those prescribed DoxyPEP, the rate of chlamydia after DoxyPEP was 91% lower than the period prior (IRR = 0.086, 95% confidence interval (CI ):0.01-0.63). For early syphilis, the post-DoxyPEP rate was 68% lower than the pre-DoxyPEP rate, but this difference did not reach statistical significance (IRR=0.32, 95% CI: 0.09-1.06). The overall rate of gonorrhea events was not significantly different before or after DoxyPEP prescription (IRR = 0.59, 95% CI 0.23-1.31) (Figure 2).

**Conclusion:**

While the overall proportion of PWH receiving DoxyPEP was low, a decreased incidence of chlamydia and possibly syphilis was observed in the 6 months after DoxyPEP prescription. Continued monitoring of real-world uptake and effectiveness will be critical during expansion of DoxyPEP implementation.

**Disclosures:**

Kaitlyn Reasoner, MD, Kindle Direct Publishing (Amazon): Royalties from a self-published book unrelated to topic of abstract

